# Predictive Structure and Topology of Peroxisomal ATP-Binding Cassette (ABC) Transporters

**DOI:** 10.3390/ijms18071593

**Published:** 2017-07-22

**Authors:** Pierre Andreoletti, Quentin Raas, Catherine Gondcaille, Mustapha Cherkaoui-Malki, Doriane Trompier, Stéphane Savary

**Affiliations:** Laboratoire Bio-PeroxIL EA7270, University of Bourgogne Franche-Comté, 6 Bd Gabriel, 21000 Dijon, France; Quentin.Raas@u-bourgogne.fr (Q.R.); Catherine.Gondcaille@u-bourgogne.fr (C.G.); mustapha.cherkaoui-malki@u-bourgogne.fr (M.C.-M.); doriane.trompier@u-bourgogne.fr (D.T.)

**Keywords:** ATP-binding Cassette (ABC) transporters, peroxisome, fatty acid transport, topology, predictive structure, adrenoleukodystrophy

## Abstract

The peroxisomal ATP-binding Cassette (ABC) transporters, which are called ABCD1, ABCD2 and ABCD3, are transmembrane proteins involved in the transport of various lipids that allow their degradation inside the organelle. Defective ABCD1 leads to the accumulation of very long-chain fatty acids and is associated with a complex and severe neurodegenerative disorder called X-linked adrenoleukodystrophy (X-ALD). Although the nucleotide-binding domain is highly conserved and characterized within the ABC transporters family, solid data are missing for the transmembrane domain (TMD) of ABCD proteins. The lack of a clear consensus on the secondary and tertiary structure of the TMDs weakens any structure-function hypothesis based on the very diverse ABCD1 mutations found in X-ALD patients. Therefore, we first reinvestigated thoroughly the structure-function data available and performed refined alignments of ABCD protein sequences. Based on the 2.85  Å resolution crystal structure of the mitochondrial ABC transporter ABCB10, here we propose a structural model of peroxisomal ABCD proteins that specifies the position of the transmembrane and coupling helices, and highlight functional motifs and putative important amino acid residues.

## 1. Introduction

ATP-Binding Cassette (ABC) transporters belong to a superfamily conserved from bacteria to humans [1]. With the exception of a few members, ABC transporters are transmembrane proteins whose minimal functional unit is predicted to contain two nucleotide-binding domains (NBDs) and two transmembrane domains (TMDs) [2]. In bacteria, each domain can result from the expression of independent genes and bind together post-translationally to form a functional ABC transporter as in the case of the *E. coli* nickel transporter [3]. There are also examples of fusion proteins containing either two NBDs (RbsA, the *E. coli* ribose transporter) or two TMDs (FhuB, the *E. coli* Vitamin B_12_ transporter) or fusion proteins composed of one NBD and one TMD, which are called half-transporters (MsbA, the *E. coli* lipid flippase for instance) [3]. In eukaryotes, homodimers (for instance ABCG2, a transporter involved in multidrug resistance) and heterodimers (for instance ABCB2/ABCB3—Tap1/Tap2, the transporter associated with antigen presentation) of half-transporters coexist with full-length ABC transporters, i.e., proteins composed of the four domains covalently linked (TMD-NBD-TMD-NBD) such as ABCB1 (P-glycoprotein), which is associated with multidrug resistance. Most of the ABC transporters or half-transporters present their TMD on their N-terminal end but the reverse domain organization also exists as in the ABCG subfamily. Auxiliary proteins participating in substrate binding, membrane anchoring or regulation may complete this common architecture in some cases. The sequence of the NBDs is very well conserved among the superfamily, in particular for the Walker A and B motifs and the ABC transporter signature (ATS) motifs which are all required for binding and hydrolysis of ATP. In contrast, a weak conservation exists among the TMDs. Classical TMDs comprise six transmembrane α-helices (TMHs) and a few conserved motifs such as the EAA motif present in intracellular loops (ICLs), which are mainly involved in the crosstalk between the TMDs and the NBDs. These interactions result in the tight coupling between substrate transport and ATP hydrolysis.

In mammals, based on structure and sequence homology, seven subfamilies from A to G have been distinguished. Peroxisomal ABC transporters belong to the subfamily D and correspond to three proteins called ABCD1 (ALDP) [4], ABCD2 (ALDRP) [5] and ABCD3 (PMP70) [6]. A fourth member of the subfamily D, ABCD4 (PMP69), originally identified as peroxisomal, is actually located into lysosomes and endoplasmic reticulum, the protein lacking a peroxisomal targeting signal [7,8,9]. Structurally, the ABCD proteins are half-transporters and must therefore dimerize to form a functional ABC transporter. Several experimental evidences argue for the existence of functional heterodimers [10,11]. However, homodimers seem to prevail in vivo [12,13]. We recently demonstrated that the dimers are mainly present within tetrameric or oligomeric structure [14]. A tetrameric assembly could be homomeric but could also consist in the assembly of two different homodimers. Among the numerous studies related to ABCD proteins, much focused on ABCD1, which is associated with the most frequent peroxisomal disorder called X-linked adrenoleukodystrophy (X-ALD) [15]. This complex neurodegenerative disorder is biochemically associated with increased levels of very long-chain fatty acids (VLCFA). X-ALD patients present a huge clinical variability both in the age of onset and in symptoms. There are two main forms: the childhood cerebral ALD associated with inflammatory demyelination of the central nervous system and the adult form called adrenomyeloneuropathy, characterized by a non-inflammatory slowly progressive demyelination affecting the spinal cord and peripheral nerves. Besides, there are few structure-function data as compared with the numerous studies published on the most famous ABC transporters (i.e., P-glycoprotein [16,17] or CFTR [18]) which benefit from crystallized proteins, site-directed mutagenesis studies and substrates allowing easy characterization of binding sites and translocation processes. Direct functional reconstitution of transport for peroxisomal ABC transporters remains a big challenge. However, recent studies, particularly in yeast as well as in animal, in plant or in cell models concluded to the participation of the ABCD1–3 transporters in the transport of various acyl-CoA esters across the peroxisomal membrane to allow their β-oxidation [19,20,21,22,23,24,25,26]. Accordingly, ABCD1 was suggested to transport preferentially saturated and monounsaturated CoA esters of VLCFA. ABCD2 presents overlapping substrate specificity with ABCD1 and was suggested to transport the same substrates probably with a lower affinity and/or efficiency. Nonetheless, ABCD2 is thought to be specifically dedicated to polyunsaturated fatty acids. Finally, ABCD3 was associated with transport of dicarboxylic acids, branched-chain fatty acids and bile acid precursors. In mammals, peroxisomal β-oxidation starts with the activation of fatty acid to its activated Co-enzyme A thioester by an acyl-CoA synthetase associated to the cytosolic side of the peroxisomal membrane. Protease protection assays have suggested that acyl-CoA esters but not free fatty acids bind to the TMD [27]. Recent works have suggested that the translocation of the acyl-CoA esters is tightly linked to an enzymatic process of CoA-ester hydrolysis [19,28,29]. The peroxisomal ABC transporters would release a free fatty acid that should be re-esterified inside the peroxisome before its catabolic processing. Once translocated inside the peroxisome, substrates could be re-esterified thanks to a specific synthetase (ACSL4, SLC27A2, and SLC27A4) [30] and directly delivered to specific Acyl-CoA oxidases (ACOX1 for VLCFA [31] or BrCACOX for branched-chain fatty acids and bile acid intermediates [32]). If true, it remains to identify motifs in charge of the protein-protein interactions.

Actually, many questions about the transport mechanism, the dimerization and oligomerization status, the substrate specificity and the substrate-binding pockets, or the peroxisomal targeting, remain to be answered. The lack of a clear consensus on the secondary and tertiary structure of ABCD transporters hampers efforts of scientists in the field and renders difficult any conceptualization of structure-function hypotheses based on mutations in patients or in site-directed mutations leading to protein truncations or modifications. As an example, UniProtKB annotations indicate 4 to 5 TMHs for ABCD proteins while most papers concerning these proteins talk about 6 transmembrane segments, the position of each α-helix differing from a source to another [33,34,35,36,37]. While a quasi-consensus seems to emerge for the position of TMH 1, 2, 4 and 5, the TMH 3 and 6 are either non-predicted or positioned differently depending on source. Altogether, this prompted us to reinvestigate the structure-function data available in the literature of peroxisomal ABC transporters in light of the latest knowledge of ABCD sequences in various species and of structural information obtained from crystallographic studies of mammalian ABC transporters. Using refined alignments of ABCD protein sequences and homology modeling based on the 2.85  Å resolution crystal structure of mitochondrial ABC transporter ABCB10, we propose a structural model of ABCD1 which specifies the position of the transmembrane and coupling helices, and highlights new functional putative motifs of the TMD.

## 2. Results

### 2.1. Position of Transmembrane α Helices

**Figure 1:**
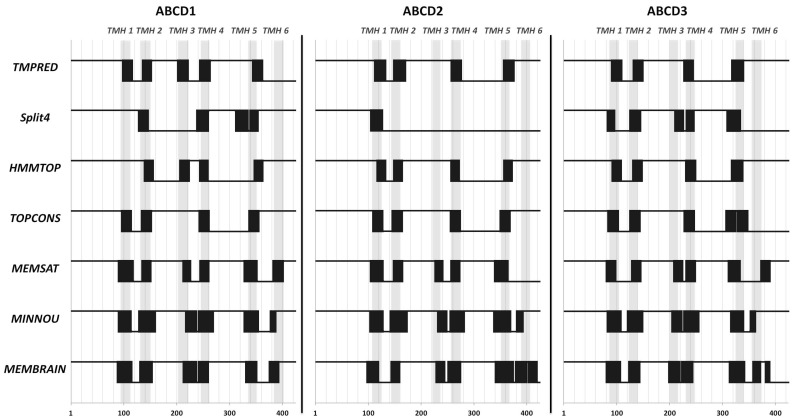
Predictive positions of the transmembrane helices (TMH) of human peroxisomal ATP-binding Cassette (ABC) transporters using various secondary structure prediction programs. Black boxes correspond to the predicted TMHs from their amino acid sequences (aa^1^–aa^425^) for each program. The grey bands correspond to the deduced position of TMHs from the refined analysis (see above).

As underlined before, many contradictory data are available on the position of the TMHs and various conserved motifs found of the ABCD proteins. Therefore, we first analyzed and compared the results of secondary structure prediction programs for ABCD1, ABCD2 and ABCD3 sequences. Accurate prediction of membrane spanning regions remains a challenge. Among the common tools available for topology prediction, TMHMM, MEMSAT, and SPLIT4 have been considered as the most robust programs but other programs such as TMpred, TOPCONS, SABLE (MINNOU server) or HMMTOP should also be considered [38,39]. The results obtained after analyses by these predictive programs are shown in Figure 1. Most of the programs display only four putative TMHs if we exclude a putative helix located close to the N-terminal end of the proteins, the helix known as the membrane peroxisomal targeting signal (mPTS) and helices found in the NBD. MemSat [40], used in the CCTOP and PSIPRED servers, as well as MINNOU [41], were able to predict 5 to 6 TMHs. MemBrain [42], a tool with improved ability in correctly identifying the ends of TMHs and which is also used in the CCTOP server, detected six to seven TMHs. While a consensus emerged for the positions of the TMH 1, 2, 4 and 5, the TMH 3 and 6 were poorly predicted. Moreover, when predicted, the different programs did not converge to a unique positioning of the TMH 3 and TMH 6.

**Figure 2:**
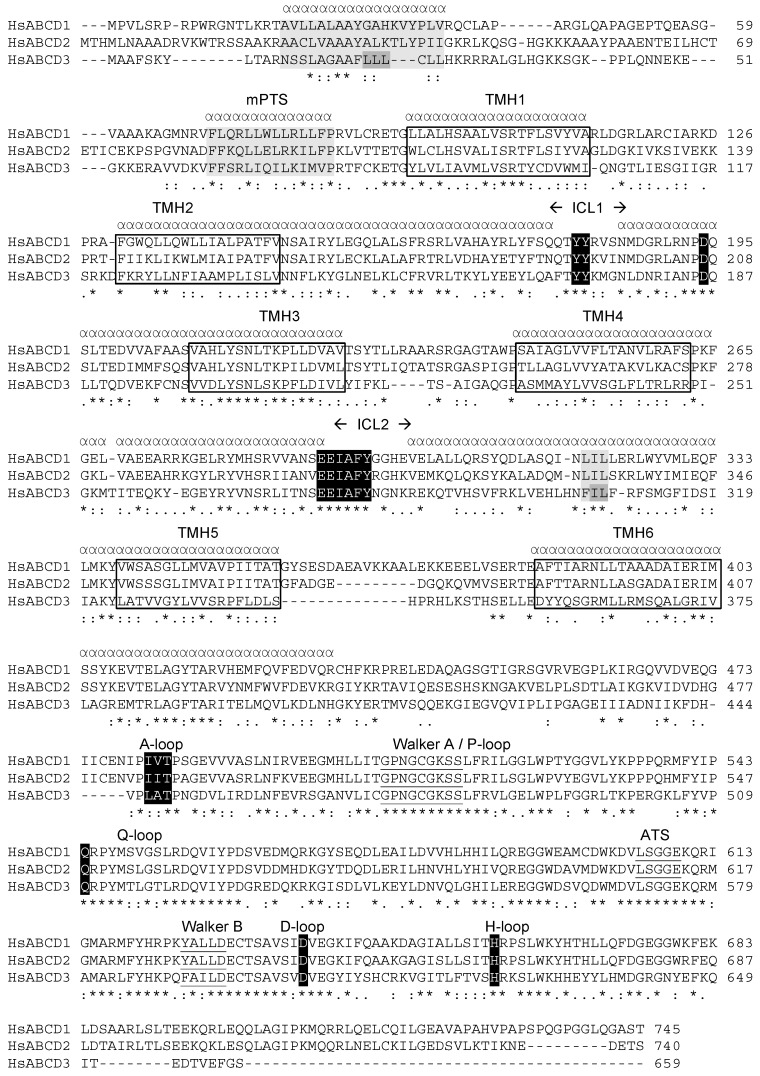
Sequence alignment of the human peroxisomal ABC transporters. Transmembrane helices (TMHs) are boxed. The membrane peroxisomal targeting signal (mPTS) in light grey is anotated. Hydrophobic clusters (containing LLL and LL motifs, dark grey) and supposed to participate in peroxisomal targeting are also in light grey. Conserved residues (“*” for identity; “:” for strong similarity; “.” for weak similarity) and helical structure (“α”) within the TMD are indicated. Intracellular loops (ICLs) 1 and 2 are indicated. Walker A and B motifs as well as the ABC-transporter signature (ATS) of the NBD are underlined. Conserved residues discussed in the text and suspected to participate in the NBD/TMD crosstalk are black boxed.

To further progress in the accurate determination of the TMHs, we performed hydrophobic cluster analysis (DRAWHCA) [43]. DRAWHCA results in a two-dimensional representation of the protein sequence, in which hydrophobic amino acids congregate into clusters, mainly corresponding to regular secondary structures [44]. A careful examination of the regions previously suspected to correspond to TMHs confirmed the positions of TMH 3 and permitted to refine the ends of the TMH 6. To finalize this analysis and to obtain the most accurate annotation of TMHs, we compared the results of various programs dedicated to membrane protein alignment (MAFFT, KALIGN, AlignMe) with local alignment of the predicted TMHs. Using Aliview, an editing software of multiple sequence alignment (MSA) based on Muscle [45], we finally obtained a refined alignment of human, mouse and rat sequences of ABCB10, ABCD1, ABCD2 and ABCD3 (Figure 2) and deduced the very likely positions of the TMHs (Table 1). Conserved residues located between the TMHs are known features of ABC transporters and emerged from this alignment reinforcing its validity. The analysis of the structural alignment computed by Espript server [46] also revealed conserved coupling helices extending the helical structure of TMHs in ABCD proteins in agreement with other ABC transporters.

### 2.2. Structural Model of ABCD1 Based on ABCB10 Homology

**Figure 3:**
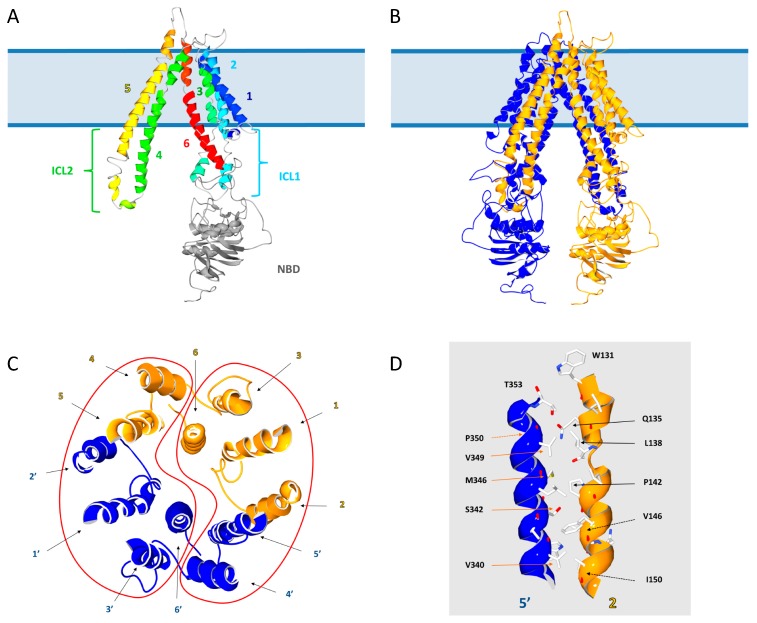
Structural model of human ABCD1. (**A**) Ribbon representation of the ABCD1 monomer. TMD helices are numbered from 1 to 6 and rainbow colored from dark blue to red. NBD is in light grey; (**B**) Ribbon representation of the ABCD1 homodimer with the two subunits respectively colored in dark blue and yellow; (**C**) Cytoplasmic view of a 32 Å cross-section of the ABCD1 TMD (with the two subunits respectively colored in dark blue and yellow) showing the TMHs organization. Transmembrane helices of each subunit are numbered from 1 to 6 for one monomer (yellow) and from 1′ to 6′ for the second monomer (dark blue). Red lines contour the two putative “supra-domains” at the interface of which the substrate could access the center of the TMD; (**D**) Ribbon representation of hydrophobic interactions between TMH 2 (in yellow) and TMH 5′ (in dark blue), sidechains of interacting amino acids are shown in CPK colored sticks. The PDB file of the model and the alignment of ABCD1 with ABCB10 are available in supplementary material and in Appendix A (Figure A1) respectively.

**Figure A1:**
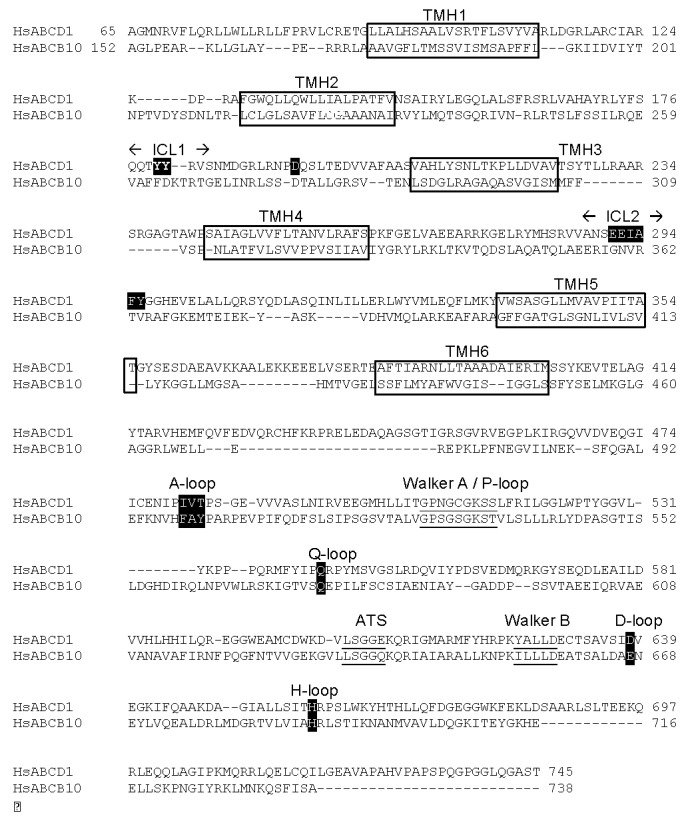
Sequence alignment of the human peroxisomal ABC transporter ABCD1 and the mitochondrial ABC transporter ABCB10. Transmembrane helices (TMHs) are boxed. Intracellular loops (ICLs) 1 and 2 are indicated. Walker A and B motifs as well as the ABC-transporter signature (ATS) of the NBD are underlined. Conserved residues discussed in the text and suspected to participate in the NBD/TMD crosstalk are black boxed.

Modelization was focused on ABCD1 only because ABCD1 is considered as the prototype of the ABCD subfamily. Moreover, ABCD1 is associated with the most frequent peroxisomal disorder (X-ALD) and many known mutations could be linked to stability, dimerization or functional concerns. In mammals, the mouse ABCB1 [17,47], as well as the human ABCC7 [18] and ABCB10 [48] proteins have been crystallized in different states. These structural data provide a reference for other ABC transporters. Among these proteins, ABCB10 displays the better percentage of identity (18%) and similarity (30%) with ABCD1. Moreover, ABCB10, like ABCD1, is a half-transporter with intracellular localization (the inner membrane of the mitochondria) and was therefore chosen as a template for structural modelization. A 2.85  Å resolution crystal structure has been determined in the presence of a non-hydrolysable analog of ATP and demonstrated an open-inwards conformation. We used the rod form of this crystal structure (PDB ID 4AYX) as a template to obtain a structural model of human ABCD1 using the Swiss-Model server (Figure 3). The structural model was constructed from the ABCD1/ABCB10 pairwise alignment presented in Appendix A (Figure A1). In order to improve the quality of the alignment, the N-terminal part of each sequence containing specific subcellular targeting signals was deleted. Gaps, mainly located in the loops linking helices, were inserted to fit with the results of the predictive secondary structures for each protein. If low confidence remains in the regions corresponding to these gaps, the overall model resulted in a reliable predictive structure, particularly in TMHs, NBDs and contact regions between ICLs and TMDs.

## 3. Discussion

Several members of the ABCD subfamily have been reported (or are predicted to be present) in a large variety of organisms and permitted to obtain a phylogenetic tree from orthologous sequences of peroxisomal ABC transporters of vertebrates (mammals, birds, fishes), insects, protozoa and nematodes, yeasts and plants [15]. These sequences were aligned and compared to highlight the conserved motifs of the TMDs and NBDs [15]. In the light of the readjusted positions of the TMHs and of the structural model presented here, the likely participation of some conserved residues in dimerization, peroxisomal targeting, and transport mechanism, is discussed below.

### 3.1. The Membrane Peroxisome Targeting Signal and the PEX19 Binding Site

From the N-terminal end of HsABCD1, the first conserved motif is located 10 amino acids before TMH 1 and corresponds to the sequence FLQRLLWLLRLLFP (71–84). The underlined residues display the highest level of conservation among the ABCD subfamily. Their conservation level reaches almost 100% if similarity is taken into account ([FAVL], [RK][LVI], [PAS]) This motif has been described as the mPTS and must be structurally followed by TMH 1 and TMH 2 to be fully effective [36]. Various truncations or deletion mutants contributed to demonstrate the direct involvement of this motif in peroxisomal targeting of ABCD1. Studies of ABCD3 led to the same conclusions [49]. Peroxisomal targeting of ABCD3 seems to depend on the presence of this motif, the TMH 1 and 2, and also on several residues located in the first 60 amino acids (LLL, 21–23) and near the TMH 5 (IL 307–308) (Figure 2) [35,50]. Interestingly, the LLL motif likely belongs to a hydrophobic helix, which is quite well conserved in other ABCD transporters (20–36 in ABCD1). Moreover, the IL motif near the TMH 5 belongs to a consensus sequence composed of three hydrophobic residues [FL]-I-[FL] (LIL in ABCD1). The conservation of these hydrophobic clusters suggests functional conservation among peroxisomal ABC transporters. Peroxisomal biogenesis factor 19 (Pex19) has been proposed to act as a chaperone for peroxisomal membrane protein, which is necessary for their integration in the peroxisomal membrane [51,52,53]. The mPTS motif was proposed to directly interact with PEX19 [54] but other studies concluded that the mPTS motif is not essential for the interaction [49]. However, interaction of ABCD3 with Pex19 would not only depend on the mPTS motif but also on the LLL and IL motifs [35,50].

### 3.2. The Intracellular Loops

From the structural data available on the ABC family, TMHs are prolonged by cytosolic amphipathic helices [55,56]. By analogy with prokaryotic ABC importers, two cytosolic helices connected by a loop form an intracellular loop (ICL), and participate in the crosstalk between the TMDs and the NBDs. In ABCD1, the putative ICL1 (between TMH 2 and TMH 3) covers 56 residues and shows very well conserved residues and motifs: Y153, LALSFRSRL (158–166), Y174, YY (180–181), RLRNPDQSLTED (189–200) (Figure 2). The role of these amino acids in peroxisomal ABC transporters is currently unknown but mutations of L158, L160, R163, R165, L166, Y181, R189, L190, P193, D194, Q195, L197, T198, E199, D200 have been described in X-ALD patients, supporting a functional key role (http://www.x-ald.nl/) [33]. Among the conserved residues of the ICL1, R163, R189, L190 and D194 are strikingly conserved in other ABC subfamilies. They correspond to R248, R274, L275, and D278 in human ABCB10 and to R148, R174, L175, and D178 in human ABCB1 ,respectively. In ABCB1, the segment between R148 and D178 is thought to play a key functional role [57]. R148 and D178 are suggested to take part in the structural stabilization of the protein and the transport mechanism. R174 would be involved in the interactions with the ICL2. F163 and D164 would interact with the intracellular helix extending the TMH 6. W162 and L171 would interact with the NBD, in particular with the Q loop. The YY 180–181 motif of ABCD1 could correspond to the aromatic residue doublet (FF in ABCB10 or WF in ABCB1) proposed to be in close contact with the NBD in ABCB1 [58]. In the closed conformation of ABCB1, the D177 residue located in the coupling helix before TMH 3 was predicted to be very close (4.4 Å) to the N820 residue of the coupling helix before TMH 9 [59]. D178 of ABCB1 is aligned with D194 in ABCD1. In the ABCD1 structural model, D194 is orientated towards the center of the TMD and was found to form a hydrogen bond with R189. Substitutions of D194 by either H or N have been described in X-ALD patient (http://www.x-ald.nl/). The ABCD1 protein bearing this D194H mutation was correctly targeted into the peroxisomal membrane. However, such mutated ABCD1 protein was shown to be non-functional and triggered a dominant negative effect on WT ABCD1 protein in situation of coexpression assay [60]. Interestingly, the equivalent mutation in ABCD2 D207H produced similar results [11].

The ICL2 (intracellular loop between TMH 4 and TMH 5 containing the EAA motif) of ABCD1 contains highly conserved amino acids as well: G266, G277, RYMHSRVVANSEEIAFYGGHEVE (280–302), Y310, L313, IL 320-321, R324 (Figure 2). Mutations have been identified for almost all these conserved positions (G266, G277, R280, H283, S284, R285, E291, E292, A294, F295, Y296, G298, E302, L313). Interestingly, the substitution G266R is one the most frequent mutations found in X-ALD patients (http://www.x-ald.nl/).

**Figure 4:**
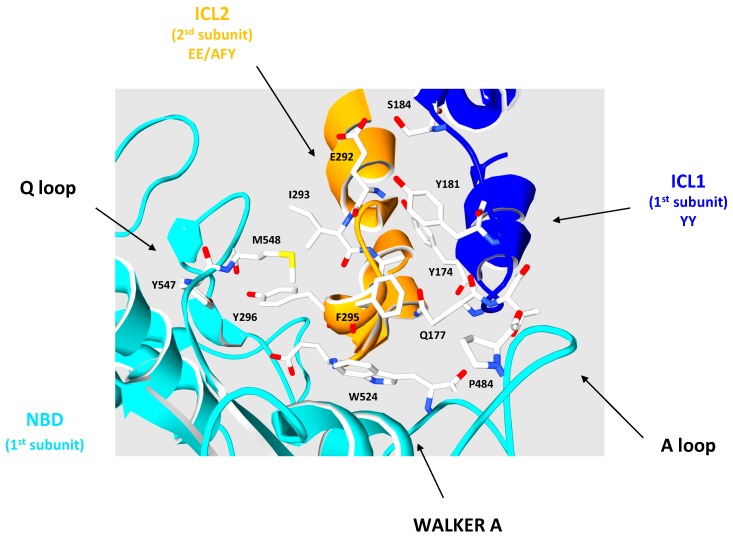
Interactions between intracellular loops (ICLs) and nucleotide-binding domains (NBDs) in the ABCD1 model. The picture shows a zoom on the interaction between the 1st subunit ICL1 (ribbon in dark blue) and the NBD (ribbon in light blue) and ICL2 (ribbon in orange) of the 2nd subunit. Amino acids involved in the interactions are shown in stick representation CPK colored. A loop, Walker A and Q loop of the NBD are arrowed.

As shown in Figure 4, a zoom in the ABCB10 structural homology model of ABCD1 permitted to precise the putative interactions between the ICLs and the NBDs. The ICL1 and the NBD of one ABCD1 subunit (respectively in dark and light blue) interact on each side of the ICL2 of the second subunit. In this model hydrophobic interactions and hydrogen bonds link the Q-loop of the NDB to one side of the ICL2 (between Y296 and Y547/M548, and between I293 and M548). The other side of the ICL2 makes close contacts with the ICL1: hydrophobic interactions between Y174 and a cluster composed of V287, L303, and L306 (buried in the inner part of the ICL2, not shown), hydrogen bonds and hydrophobic interactions between Y181 and S184 and E292. A hydrophobic interaction links the ICL1 and the NDB A loop (respectively Q177 and P484). Finally, a clear hydrophobic interaction between F295 and W524 participates in the interaction between ICL2 and the NBD P-loop, respectively.

As underlined by Beek et al. [61] and contrarily to importers, ABC exporters (such as ABCD1) contain a second coupling helix located in ICL1 between TMH 2 and TMH 3. As said above, the role of these helices is to transduce signals from TMD to NBD to allow ATP binding and hydrolysis and from NBD to TMD to carry out the substrate transport through conformational changes. So, the existence of these two coupling helices, from two different subunits, involving four TMHs of the TMD (TMHs 2 to 5), with strong interaction between them and with the NDB, suggests the need for a global conformational modification to perform the substrate translocation.

### 3.3. Motifs Putatively Involved in Substrate Specificity

The role of peroxisomal ABC transporters has been ascribed to the transport of different substrates: acyl-CoA esters of saturated and monounsaturated VLCFA for ABCD1, polyunsaturated LCFA and VLCFA for ABCD2, branched-chain FA, dicarboxylic FA and bile acid precursors for ABCD3. Previous studies on ABC transporters involved in multidrug resistance [62] revealed that the substrate preference of peroxisomal ABC transporters depends on binding sites located in the TMHs. The poor sequence conservation in the TMDs is in agreement with the diversity of lipids displaying various sizes, physicochemical and biophysical properties that have to interact with these ABC transporters. However, a partial functional redundancy between ABCD1, ABCD2 and ABCD3 has been documented and is probably associated with some conserved residues of the TMHs, particularly in the TMH 1, 2 and 3 (Figure 2). The lack of adequate assays to reconstitute substrate transport by ABCD transporters renders difficult the identification of substrate binding pockets and interaction motifs within the TMHs. Since the substrates are predicted to contact the TMHs through the membrane interface, they must pass between TMHs to reach the core of the TMD. Our model presents strong hydrophobic interactions between helices 2 and 5′ (and 2′ and 5) of the two monomers (Figure 3D). The access to the inner part of the TMD through the monomers interface could thus be energetically difficult. It is to note that interactions between TMH 3 and TMH 4, and between TMH 4 and TMH 6 appear less tight and could be a way for substrates to enter the TMD (Figure 3C). Consequently, the TMD could be divided in two “supra-domains” containing each six TMHs: one supra-domain with TMHs 1′, 2′, 3′, 4, 5 and 6’ and the second supra-domain with TMHs 1, 2, 3, 4’, 5’ and 6 (contoured by a red line in Figure 3C), the substrate accessing the center of TMD through the interface of these two “supra-domains”.

In this quest towards the identification of a substrate-binding site, the hydrophobic nature of substrates should be taken into account. Protein-protein interactions are proposed to play an important role in the transport mechanism, first to permit the substrate to reach the peroxisomal membrane and then to be released inside the peroxisome. This would suppose protein-protein interactions involving transmembrane helices, cytosolic loops as well as peroxisomal internal loops and a tight incidence between these interactions and the substrate specificity. Specific fatty acid binding proteins and synthetases have been proposed to bind and activate fatty acids and release the substrates, i.e., acyl-CoAs, to the ABC transporters [30]. These substrate modifications could be tightly associated with the transport mechanism [19,28,29]. If true, protein-protein interactions between these accessory proteins and the ABC transporters are required. There are 3 loops proposed to be located in the lumen of the peroxisome (Figure 2 and Figure 3A). The loop between TMH 5 and TMH 6 being the longest one and corresponding to one of the less conserved zones of the TMD, both in size and in sequence, we postulate a possible role in such interactions. Interestingly, missense mutations in X-ALD patients have been described in this region but they have unfortunately not been studied in more detail.

### 3.4. Motifs Putatively Involved in Dimerization

Dimerization is considered as an obligatory step for stability and function of peroxisomal ABC transporters. If we recently evidenced that these proteins form mainly tetramers [14], there are currently no evident motifs responsible for such interactions. The amino acids responsible for the interactions within a dimer remain to be characterized too. Dimerization probably depends on residues belonging to the NBD [63,64]. However, as said before, our structural model highlights a close proximity between the TMH 2 of the first subunit and the TMH 5 of the second one (TMH 5′, Figure 3C), proximity that is prolonged through the coupling helices. A zoom in that region suggests the existence of multiple hydrophobic interactions involving W131/T353, Q135/P350, L138/V349, P142/M346, V146/S342, I150/V340. Further studies will be needed to confirm such interactions.

## 4. Methods

### 4.1. Prediction of Transmembrane Helices

Several programs were used to predict the TMHs of peroxisomal ABC transporters: TMpred, SPLIT4, HMMTOP, TOPCONS, MEMSAT, MINNOU and MemBrain [38,39]. Hydrophobic cluster analysis (DRAWHCA) was used as a complementary analysis [43].

### 4.2. Alignment

Aliview, an editing software of alignment based on Muscle algorithm [45] was used to perform MSA of peroxisomal ABC transporter sequences. Alignment of human ABCD1 with human ABCB10 was performed in three steps. First, the human, mouse and rat sequences of ABCB10, ABCD1, ABCD2 and ABCD3 were aligned. Then, the known positions of the TMHs of ABCB10 were used as anchors to perform local alignment with the putative TMHs of the ABCD proteins. Finally, rodent sequences were removed as well as ABCD2 and ABCD3 sequences in order to obtain an ACBD1/ABCB10 pairwise alignment suitable for homology modeling.

### 4.3. Homology Modeling

Homology modeling was computed using the “target-template alignment” mode of the SWISS-MODEL [65,66,67]. The optimized ABCD1/ABCB10 pairwise alignment (Figure A1) computed was used as template to direct the homology modeling process against the 2.85 Å resolution rod form structure of ABCB10 (PDB ID 4AYX). A sequence identity of 17.7% for the alignment was sufficient to Swiss-Model server to successfully compute a homodimeric model for ABCB1 (pdb file in Supplementary Data).

## 5. Conclusions

In conclusion, the present study provides a refined TMH annotation and a structural model of ABCD1, which might help structure-function investigations on the impact of ABCD1 mutations associated with X-ALD. Regarding the sequence homology between ABCD1 and ABCD2 or ABCD3, the hypotheses raised by this study should apply at the other peroxisomal ABC transporters. However, given the limitations of the structural model (low similarity between ABCB10 and ABCD1), further structural data will be required to achieve better reliability and complete elucidation will only be obtained after protein purification and crystallographic analysis of peroxisomal ABC transporters.

## Figures and Tables

**Table 1:** Deduced positions of the transmembrane helices in human peroxisomal ATP-binding Cassette transporters

Transporter	TMH 1	TMH 2	TMH 3	TMH 4	TMH 5	TMH 6
ABCD1	93–112	130–147	208–224	244–262	338–355	384–403
ABCD2	106–125	143–160	221–237	257–274	351–368	388–407
ABCD3	85–104	122–139	200–216	231–248	324–341	356–375

## Supplementary Materials

Supplementary materials can be found at www.mdpi.com/1422-0067/18/7/1593/s1.

## Abbreviations

ABC	ATP-binding Cassette
ATS	ABC Transporter Signature
ICL	IntraCellular Loop
FA	Fatty Acid
MSA	Multiple Sequence Alignment
NBD	Nucleotide-binding Domain
TMD	TransMembrane Domain
TMH	TransMembrane Helix
VLCFA	Very Long-Chain Fatty Acid
X-ALD	X-linked Adrenoleukodystrophy
